# The neocortical microcircuit collaboration portal: a resource for rat somatosensory cortex

**DOI:** 10.3389/fncir.2015.00044

**Published:** 2015-10-08

**Authors:** Srikanth Ramaswamy, Jean-Denis Courcol, Marwan Abdellah, Stanislaw R. Adaszewski, Nicolas Antille, Selim Arsever, Guy Atenekeng, Ahmet Bilgili, Yury Brukau, Athanassia Chalimourda, Giuseppe Chindemi, Fabien Delalondre, Raphael Dumusc, Stefan Eilemann, Michael Emiel Gevaert, Padraig Gleeson, Joe W. Graham, Juan B. Hernando, Lida Kanari, Yury Katkov, Daniel Keller, James G. King, Rajnish Ranjan, Michael W. Reimann, Christian Rössert, Ying Shi, Julian C. Shillcock, Martin Telefont, Werner Van Geit, Jafet Villafranca Diaz, Richard Walker, Yun Wang, Stefano M. Zaninetta, Javier DeFelipe, Sean L. Hill, Jeffrey Muller, Idan Segev, Felix Schürmann, Eilif B. Muller, Henry Markram

**Affiliations:** ^1^Blue Brain Project, École Polytechnique Fédérale de Lausanne (EPFL) Biotech CampusGeneva, Switzerland; ^2^Department of Neuroscience, Physiology and Pharmacology, University College LondonLondon, UK; ^3^CeSViMa, Centro de Supercomputación y Visualización de Madrid, Universidad Politécnica de MadridMadrid, Spain; ^4^Laboratory of Neural Microcircuitry, Brain Mind Institute, École Polytechnique Fédérale de LausanneLausanne, Switzerland; ^5^Key Laboratory of Visual Science and National Ministry of Health, School of Optometry and Opthalmology, Wenzhou Medical CollegeWenzhou, China; ^6^Caritas St. Elizabeth's Medical Center, Genesys Research Institute, Tufts UniversityBoston, MA, USA; ^7^Laboratorio Cajal de Circuitos Corticales, Centro de Tecnología Biomédica, Universidad Politécnica de MadridMadrid, Spain; ^8^Instituto Cajal (CSIC) and CIBERNEDMadrid, Spain; ^9^Department of Neurobiology, Alexander Silberman Institute of Life Sciences, The Hebrew University of JerusalemJerusalem, Israel; ^10^The Edmond and Lily Safra Centre for Brain Sciences, The Hebrew University of JerusalemJerusalem, Israel

**Keywords:** neocortex, microcircuit, models, experimental data, morphologies, neurons, ion channels, synapses

We have established a multi-constraint, data-driven process to digitally reconstruct, and simulate prototypical neocortical microcircuitry, using sparse experimental data. We applied this process to reconstruct the microcircuitry of the somatosensory cortex in juvenile rat at the cellular and synaptic levels. The resulting reconstruction is broadly consistent with current knowledge about the neocortical microcircuit and provides an array of predictions on its structure and function. To engage the community in exploring, challenging, and refining the reconstruction, we have developed a collaborative, internet-accessible facility—the Neocortical Microcircuit Collaboration portal (NMC portal; https://bbp.epfl.ch/nmc-portal). The NMC portal allows users to access the experimental data used in the reconstruction process, download cellular and synaptic models, and analyze the predicted properties of the microcircuit: six layers, ~31,000 neurons, 55 morphological types, 11 electrical types, 207 morpho-electrical types, 1941 unique synaptic connection types between neurons of specific morphological types, predicted properties for the anatomy and physiology of ~40 million intrinsic synapses. It also provides data supporting comparison of the anatomy and physiology of the reconstructed microcircuit against results in the literature. The portal aims to catalyze consensus on the cellular and synaptic organization of neocortical microcircuitry (ion channel, neuron and synapse types and distributions, connectivity, etc.). Community feedback will contribute to refined versions of the reconstruction to be released periodically. We consider that the reconstructions and the simulations they enable represent a major step in the development of *in silico* neuroscience.

## Introduction

The Blue Brain Project has developed a unifying process for the pragmatic integration of available data and knowledge into *in silico* reconstructions of neuronal microcircuits. We have used this process to reconstruct the microcircuitry of developing rat [postnatal days (P) 13–16] somatosensory cortex from sparse data on its cellular and synaptic organization (Markram et al., 2015). The resulting reconstruction is made up of component data covering multiple levels of detail: ion channels, synapses, neurons, and the entire microcircuit. The reconstruction evolves in successive cycles of reconstruction-validation-experiment-reconstruction, and will continuously integrate new properties and principles as they become available.

The anatomy and physiology of the reconstruction can be studied in the same way as those of a block of neocortical tissue. To enable such studies, we have developed an internet-accessible public resource—The NMC Portal. The portal provides access to the data, literature, and models used in the reconstruction of P13-16 rat somatosensory cortex, together with interactive tools to browse and query its detailed anatomy and physiology. The resource also provides means for the community to explore, analyze, and annotate the properties of the reconstruction, to compare them against results from the literature, and to provide feedback for future releases.

## The reconstructed neocortical microcircuit

The reconstructed microcircuit represents six cortical layers with an overall thickness of ~2.1 mm, and a volume of ~0.29 mm^3^. It is constituted by ~31,000 neurons belonging to 55 morphological types (m-types), 11 electrical types (e-types), and 207 morpho-electrical types (me-types); ~7.5 million connections belonging to 1941 unique m-type specific connection types; and ~40 million synapses belonging to six synapse types (s-types) (Markram et al., 2015). The overall dimensions, individual layer thicknesses, and the ratio of excitatory to inhibitory neurons in the reconstruction are consistent with results from previous studies of rat barrel cortex (Meyer et al., 2010, 2011; Wimmer et al., 2010). However, the total number of neurons in the reconstruction based on measurements in juvenile (P13-16) rat somatosensory cortex is around 1.5 times higher than in adult (P27) rat barrel cortex (Meyer et al., 2010).

## The NMC portal—an overview

The portal allows users to access the experimental data used in the reconstruction process, download cellular and synaptic models, and analyze the predicted properties of the microcircuit. The portal is structured into the following sections (see Supplementary Figure 1):

### Microcircuit

An interactive browser across three levels: *layers, neurons*, and *synapses*. The *layer* level provides data on thickness, neuron, and synapse densities, and the distributions of neuron and synapse types for each of the six layers of the reconstruction. The *neuronal* level describes the anatomical and physiological properties of morphological, electrical and morpho-electrical neuron types (m-, e-, and me-types, respectively). The *synaptic* level represents the anatomical and physiological properties of synaptic connections between specific pre-post combinations of m-types, and the complete map of intrinsic input and output synapses (three excitatory, and three inhibitory synaptic types), from and to neurons of different types.

### Literature consistency

Comparisons between the morphological, molecular, electrical, synaptic, and physiology properties of the reconstruction and its overall circuit anatomy against the published literature.

### Experimental data

Experimental datasets used in the reconstruction process.

### Videos

Animations of simulated microcircuit activity under a variety of experimental conditions.

### Images

Images illustrating key steps in the reconstruction process.

### Tools

Tools for analyzing, and simulating the reconstructed microcircuit.

### Downloads

Models of neurons, ion channels, and synapses for the NEURON simulation environment.

## The microcircuit

The microcircuit section of the portal contains data computed during the reconstruction process, using methods described in (Markram et al., 2015). The data is presented in fact sheets, each representing a different level of biological organization.

**Figure 1:**
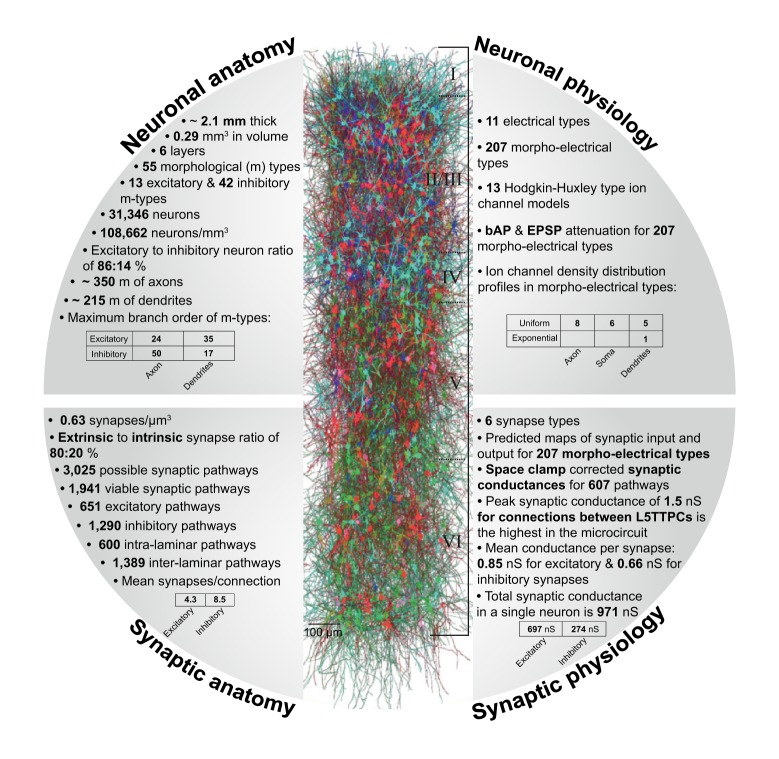
**An overview of the reconstructed microcircuit—facts and figures. Top left:** facts and figures on the neuronal anatomy of the reconstructed microcircuit. **Top right:** overview of neuronal physiology. **Bottom left:** facts and figures on synaptic anatomy. **Bottom right:** overview of synaptic physiology.

The **fact sheet** for the whole reconstructed microcircuit provides an integrated view of its dimensions, layer-wise distributions and densities of neurons, total number of morphological types (m-types), electrical types (e-types), morpho-electrical types (me-types), and synapse types (s-types), numbers of intrinsic, and extrinsic synaptic connections and associated synapses, and the number of unique synaptic connection types between neurons of specific source and target m-types (see Figure 1).

### Layers

**Figure 2:**
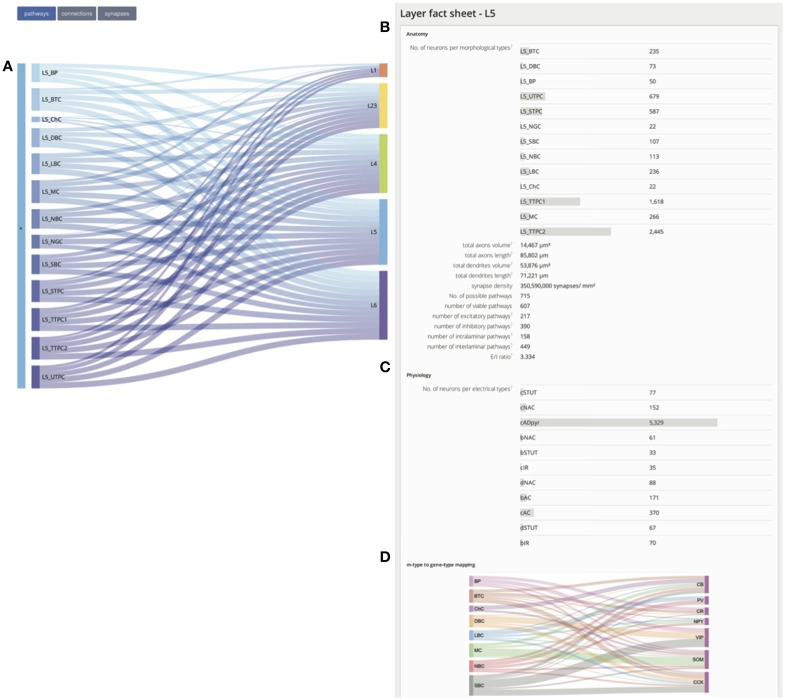
**Layer fact sheet. (A)** The pathway navigator predicting the post-synaptic target layers of all m-types in layer 5. The thickness of a connecting ribbon indicates the number of possible pathways (connections or synapses, based on the user selection in the top left) of a given layer 5 m-type to the recipient layer. **(B)** Overview of the m-type composition in layer 5, morphometrics, and synaptic anatomy. **(C)** Proportional composition of different e-types in layer 5. **(D)** Map of commonly expressed calcium binding proteins and neuropeptides within inhibitory neurons of different m-types in layer 5. The thickness of the connecting ribbon indicates the prevalence of specific markers.

The **layer level fact sheets** (see Figure 2) provide a unified picture of the anatomical and physiological properties of each layer in the reconstructed microcircuit as outlined below.

1. The total number of neurons in each designated layer and for each morphological type (m-type) in a layer are given (see Figure 2B), where the naming of m-types was based on the most common names used in previous studies (Larkman, 1991; Kawaguchi and Kubota, 1997; DeFelipe et al., 2002; Wang et al., 2006; Lübke, 2003; Ascoli et al., 2008; Romand et al., 2011).
2. Total axonal length and volume, total dendritic length and volume, synapse density, total number of morphology-specific synaptic connections are provided, and categorized into: geometrically possible and viable pathways, excitatory and inhibitory pathways, and intra- and inter-laminar pathways (Thomson and Deuchars, 1997; Somogyi et al., 1998; Feldmeyer et al., 1999, 2002; Gupta et al., 2000; Wang et al., 2002; Silberberg and Markram, 2007).
3. The proportion of different electrical types (e-types) for each layer is given (see Figure 2C for an example of e-type proportions in layer 5).
4. The mapping of inhibitory m-types to the main calcium-binding proteins [parvalbumin (PV), calbindin (CB), and calretinin (CR)], and neuropeptides [somatostatin (SOM), vasoactive intestinal polypeptide (VIP), neuropeptide Y (NPY), and cholecystokinin (CCK)] they express is given (see Figure 2D) as described previously (DeFelipe, 1993; Gonchar and Burkhalter, 1997; Kawaguchi and Kubota, 1997; Kawaguchi and Kondo, 2002; Toledo-Rodriguez et al., 2005; Gonchar et al., 2008; O'Connor et al., 2009; Meyer et al., 2011; Santana et al., 2013).

### Morphological types (m-types)

**Figure 3:**
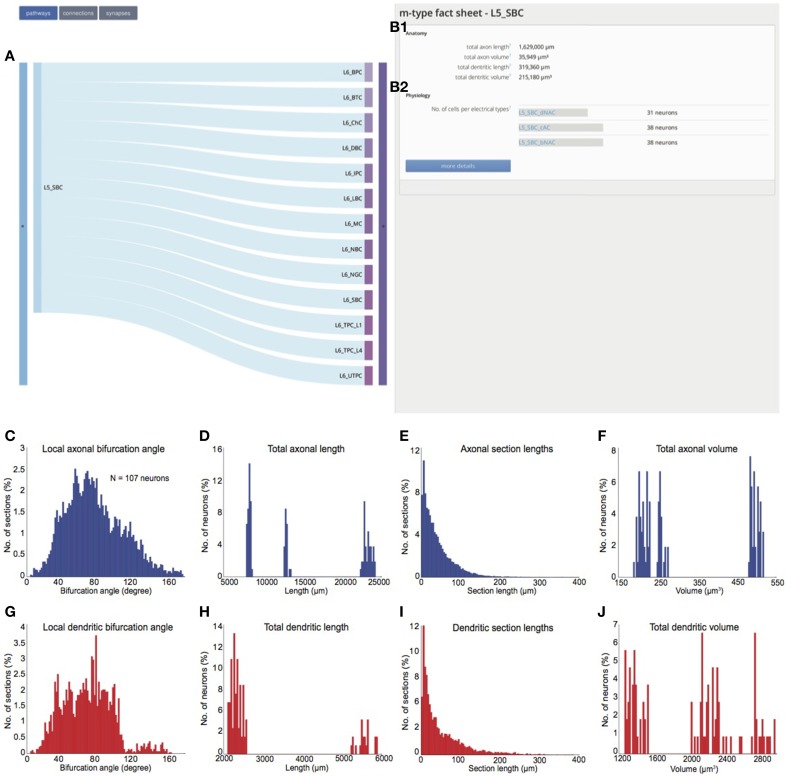
**m-type fact sheet. (A)** Predicted post-synaptic targets for neurons belonging to the L5_SBC m-type; **(B1)** anatomical fact sheet for all neurons of the L5_SBC m-type. **(B2)** e-types expressed in neurons belonging to the L5_SBC m-type **(C)** Histogram of axonal section bifurcation angles compiled from all reconstructed L5_SBC morphologies (*N* = 106). **(D)** Histogram of total axonal lengths. **(E)** Histogram of individual axonal section lengths. **(F)** Histogram of axonal volumes. **(G)** Histogram of dendritic section bifurcation angles. **(H)** Histogram of total dendritic lengths. **(I)** Histogram of individual axonal section lengths. **(J)** Histogram of dendritic volumes.

The **m-type fact sheets** (see Figure 3) provide an anatomical overview of total axonal and dendritic lengths, and volumes for individual m-types (see Figure 3B1). Previous studies have established that neurons of particular m-types display diverse electrical behavior (Kawaguchi and Kubota, 1997; Gupta et al., 2000; Markram et al., 2004; Toledo-Rodriguez et al., 2004). Therefore, the m-type fact sheets also list the different e-types associated with the m-type (see Figure 3B2). In addition, the fact sheets provide detailed anatomical characterizations of the axonal and dendritic properties of neurons belonging to the m-type. These include section bifurcation angles (Figures 3C,G), total lengths (see Figures 3D,H), individual section lengths (see Figures 3E,I), and volumes (Figures 3F,J).

### Morpho-electrical types (me-types)

**Figure 4:**
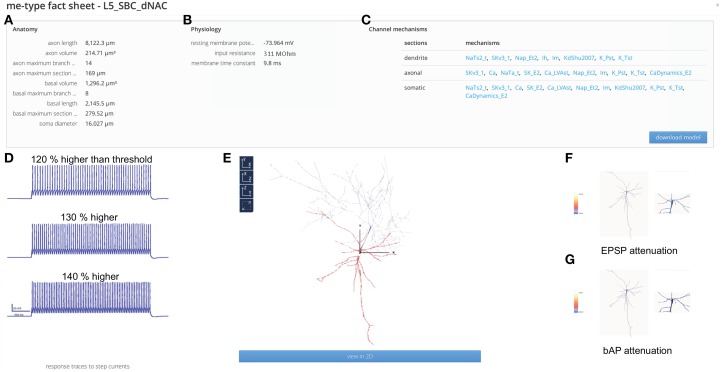
**me-type fact sheet**. **(A)** Anatomical properties of all neuron models belonging to the L5_SBC-dNAC me-type. **(B)** Physiological properties of all neuron models belonging to the L5_SBC-dNAC me-type. **(C)** Density distributions of ion channels used in neuron models. **(D)** Representative response traces in a L5_SBC_dNAC me-type model to different intensities of somatically injected step currents. **(E)** 3D reconstructed morphology of an exemplar L5_SBC neuron. **(F)** Predicted dendritic attenuation of EPSPs. **(G)** Predicted attenuation of the back-propagating AP.

The **me-type fact sheets** provide an in-depth description of the anatomical and physiological properties of each of the 207 individual me-types in the reconstruction, and detailed data for five exemplar neurons of each me-type (Figure 4; see also Figure 4E for a single exemplar): total length and volume, maximal section length, maximal branch order, and soma diameter (see Figure 4A). The physiological characterization includes standard metrics for intrinsic membrane properties (Connors and Gutnick, 1990; Kasper et al., 1994; Zhu, 2000): resting potential, input resistance, and membrane time constant (see Figure 4B). The fact sheets also provide a list of relevant ion channels models for specific me-types, and data on the distribution of ion channels along neuronal arbors, identified from the literature (see Figure 4C) (Stuart and Sakmann, 1994; Korngreen and Sakmann, 2000; Kole et al., 2006). These data make it possible to model the electrical firing patterns of specific me-types, as shown in Figure 4D, which illustrates modeled electrical responses to somatic step current injections of varying intensities. The fact sheets link to the relevant model packages, which are designed for use in the NEURON simulation environment.

Neuronal dendrites not only funnel synaptic input toward the axon, but also sustain action potentials (AP) back-propagating from the axon (Stuart and Sakmann, 1994). However, experimental data characterizing the active dendritic properties of neocortical neurons is only available for a small subset of me-types (Larkum et al., 2007, 2009; Nevian et al., 2007; Ledergerber and Larkum, 2010). The reconstruction predicts these values. The me-type fact sheets provide predictions (movies) of dendritic back-propagating action potentials (bAP), and the attenuation of post-synaptic potentials (PSP) for all 207 me-types (see Figures 4F,G).

### Connection types

The 55 m-types constituting the microcircuit could theoretically form 3025 morphology-specific connection types. Consideration of the overlap of axons and dendrites, or lack thereof, in the reconstruction predicts that 1941 of these connection types are geometrically viable. Although, only around 1% of such connection types have been characterized experimentally (Thomson et al., 1993; Markram et al., 1997; Reyes et al., 1998; Feldmeyer, 2006), the reconstruction makes it possible to predict their properties, which are presented in connection type fact sheets. Each fact sheet shows the predicted map of synaptic connections from neurons of one m-type to neurons of other m-types (Supplementary Figure 2A), together with data for their predicted anatomy, and physiology, (Supplementary Figure 2B).

**Figure 5:**
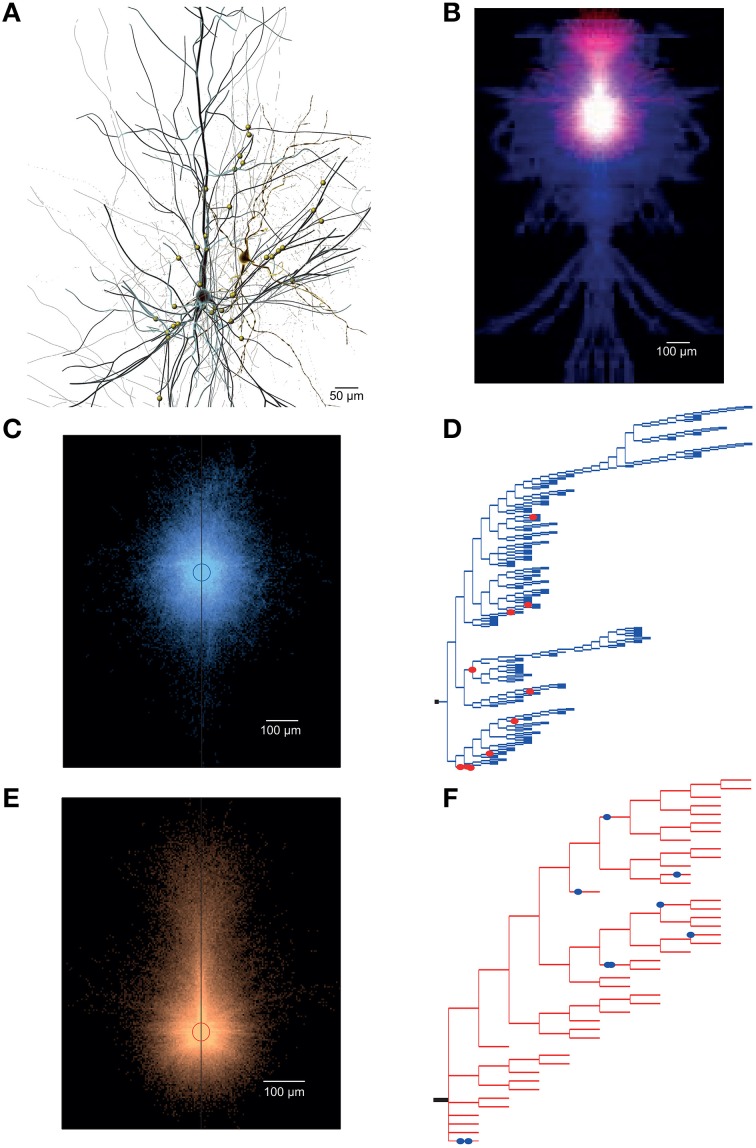
**Overview of ***in silico*** synaptic anatomy. (A)** An exemplar *in silico* pair of synaptically connected L23_SBC (right, yellow) and L23_PC (left, black) neurons. The synaptic contacts mediating the connection are shown as yellow circles on the L23_PC. **(B)** Overlap of axo-dendritic density clouds of all pre-synaptic L23_SBCs and post-synaptic L23_PCs. **(C)** Overlap of axonal density clouds of all pre-synaptic L23_SBCs. **(D)** Axogram of the pre-synaptic L23_SBC showing the location of synaptic contacts (red circles) along the axon (in blue). **(E)** Overlap of dendritic density clouds of all pre-synaptic L23_PCs. **(F)** Dendrogram of the post-synaptic L23_PC, showing the location of synaptic contacts (blue circles) along the dendrites (in red).

The anatomical data includes total synapse count, average connection probability, number of common neighbors (see Perin et al., 2011), number of convergent and divergent neurons, and the mean number of synapses per connection (synapses/connection) for all connections between pairs of neurons belonging to a specific connection type (Supplementary Figure 2B). It also provides more detailed data, including a graphical representation of an exemplar pair of synaptically connected neurons (see Figure 5A), overlapping axo-dendritic density clouds for all pre- post-synaptic neuron pairs that belong to the connection type (Figure 5B), the overlaid axonal and dendritic density clouds for individual pre-synaptic and post-synaptic morphologies (Figures 5C,E, respectively), the axogram for the morphology of the pre-synaptic neuron in the exemplar pair (as shown in Figure 5A), predicting the axonal location of synaptic contacts (Figure 5D), and the dendrogram for the morphology of the post-synaptic neuron in the exemplar pair, predicting the dendritic location of synaptic contacts (Figure 5F).

**Figure 6:**
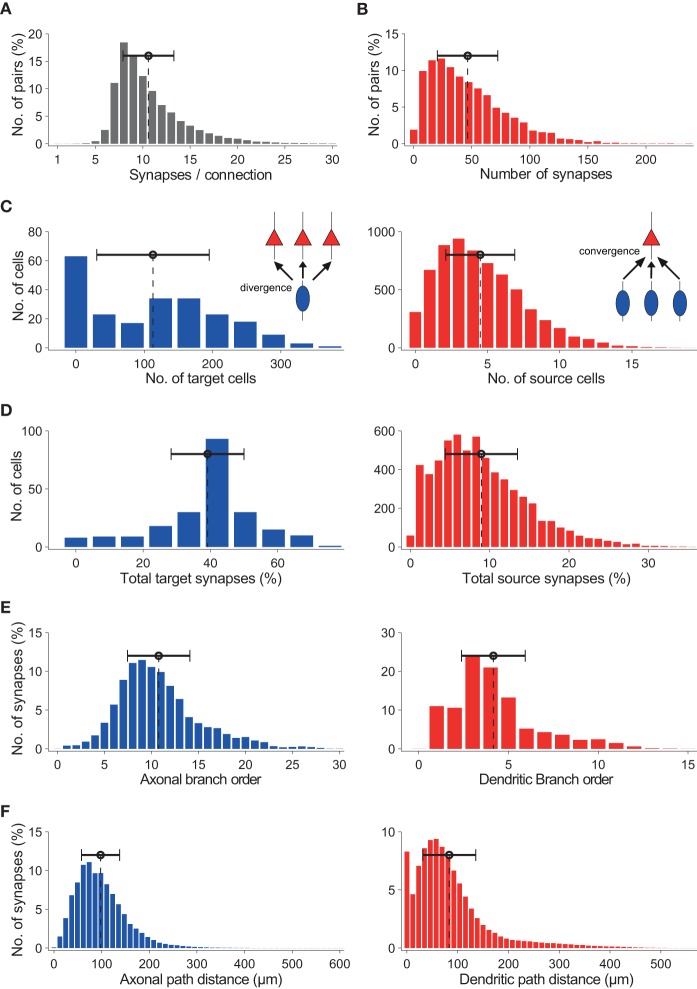
**Characterization of ***in silico*** synaptic anatomy. (A)** Distribution of number of synapses per connection for pairs of synaptically connected L23_SBC to L23_PC neurons. **(B)** Distribution of the total number of synapses from all L23_SBCs to L23_PCs. **(C)** Left: neuronal divergence; number of L23_PCs targeted by individual L23_SBCs. Right: neuronal convergence - number of L23_SBCs targeting individual L23_PCs. **(D)** Left: synaptic divergence - fraction of all synapses formed by L23_SBCs that target L23_PCs. Right: synaptic convergence; fraction of all synapses formed onto L23_PCs that originate from L23_SBCs. **(E)** Axonal (left) and dendritic (right) innervation patterns, in terms of branch order of synaptic contacts. **(F)** Same as **(E)**, but in terms of geometrical distance of synaptic contacts.

Additionally, the fact sheets provide detailed statistical distribution for the number of synapses per connection (Figure 6A), the total number of synapses from all pre-synaptic neurons of a given type to a post-synaptic neuron of a given type (Figure 6B), the number of post-synaptic neurons of a given type targeted by individual neurons of a given type (i.e., neuronal divergence; Figure 6C, left), the number of pre-synaptic neurons of a given type, targeting individual post-synaptic neurons of a given type (i.e., neuronal convergence; Figure 6C, right), the fraction of efferent synapses from a given neuron type targeting neurons of a specific type (i.e., synaptic divergence; Figure 6D, left), the fraction of afferent synapses from neurons of a specific type targeting neurons of a specific type (i.e., synaptic convergence; Figure 6D, right), patterns of axonal and dendritic synaptic innervation based on branch order (Figure 6E; left and right, respectively), and path distance from the soma (Figure 6F; left and right, respectively).

The physiological data provides predictions at the level of single synapses and connections (Supplementary Figure 2B). Each synapse is assigned to one of six s-types (Gupta et al., 2000; Wang et al., 2006). Individual synapses are characterized by their peak conductance (g_*syn*_; in nS), neurotransmitter release probability (U), time constant for recovery from depression (D; in ms), time constant for recovery from facilitation (F; in ms), and the predicted correction factor necessary to account for dendritic filtering (space-clamp artifacts). Physiological data at the connection level summarizes the kinetics and time course of post-synaptic potentials (PSPs). The data provided include onset latencies, peak amplitudes, 20–80% rise times, decay time constants, transmission failures, and the coefficient of variation of PSP amplitudes (c.v.; defined as the ratio of the standard deviation to the mean of PSP amplitude) (see Ramaswamy and Markram, 2015).

**Figure 7:**
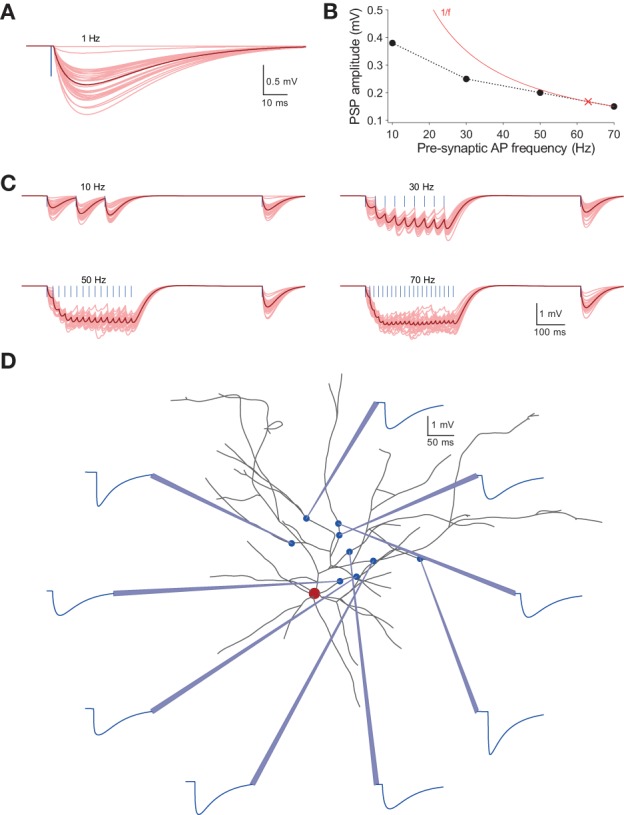
**Overview of ***in silico*** synaptic physiology. (A)** Average time course and amplitude of *in silico* unitary PSPs measured in a post-synaptic L23_PC by evoked unitary APs in a pre-synaptic L23_SBC (for the same pair of neurons shown in Figure 5A); blue line to the left indicates the pre-synaptic AP; pink traces, individual post-synaptic responses across 30 trials; red trace, average of 30 trials. **(B)** The 1/f rule demonstrating an inverse relationship between stationary PSPs and the frequency of pre-synaptic stimulation; the solid line in red shows the inverse relationship with frequency, and the cross shows the limiting frequency. **(C)** Frequency dependence of synaptic transmission. Clockwise from top left: *in silico* PSPs evoked in the post-synaptic L23_PC upon stimulating the pre-synaptic L23_SBC with pulse trains at frequencies of 10, 30, 70, and 50 Hz, respectively (for the same pair of neurons shown in Figure 5A). **(D)** The digital microcircuit reconstruction enables direct *in silico* recordings of single synaptic contacts on dendrites. For the synaptically connected pair (shown in Figure 5A), post-synaptic responses were recorded directly at synaptic locations on dendrites of the L23_PC (dendrites in black; soma in red); the connection was mediated by nine synaptic contacts (blue circles); dendritic recording sites are shown as light blue pipettes; corresponding PSPs are shown in blue.

Additionally, the fact sheets provide data on the trial-to-trial response variability of unitary PSPs for given types of post-synaptic neurons (Figure 7A for the exemplar pair shown in Figure 5A), synaptic response to pre-synaptic stimulation at 10, 30, 50, and 70 Hz (Figure 7C, clockwise from top left), and the relationship between the frequency of pre-synaptic stimulation and PSP amplitude. These data illustrate the 1/f law for synapses found in previous experimental studies [(Figure 7B); (Tsodyks and Markram, 1997); also see (Ramaswamy and Markram, 2015)]. The fact sheets also provide data from virtual recordings of local dendritic PSPs (Figure 7D). It is currently not possible to obtain comparable data through experiments alone.

**Figure 8:**
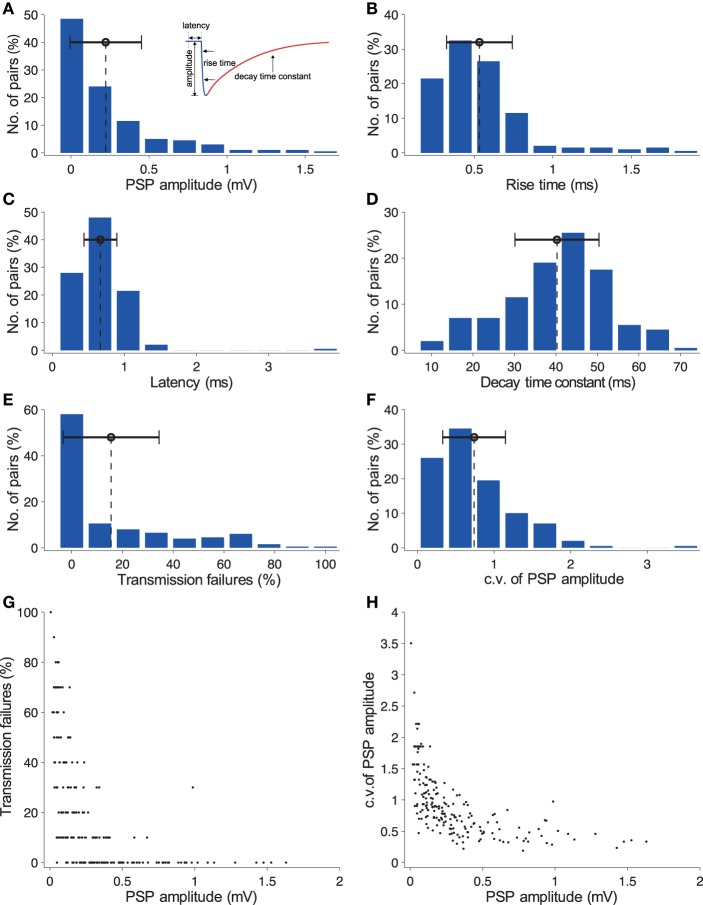
**Characterization of ***in silico*** synaptic physiology**. **(A)** Histogram of *in silico* PSP amplitudes for synaptically connected pairs of L23_SBC and L23_PC neurons (*n* = 100 pairs, sampled at inter-somatic distances ≤ 100 μm); the dashed line in black indicates the mean of the distribution; the error bar shows the standard deviation (SD). **(B)** Histogram of *in silico* 20–80% PSP rise times. **(C)** Histogram of *in silico* PSP onset latencies. **(D)** Histogram of *in silico* PSP decay time constants. **(E)** Histogram of transmission failures. **(F)** Histogram of the c.v. of PSP amplitudes. **(G)** Predicted inverse relationship between the rate of transmission failures and PSP amplitude; the reconstructed microcircuit predicted a decrease in failure rates with increasing PSP amplitudes, consistent with a binomial model of transmitter release. **(H)** Same as in **(G)**, but for c.v. of PSP amplitudes.

The fact sheets also provide detailed statistical distributions for the kinetics and average time course of PSPs, including mean PSP amplitude (Figure 8A), 20–80% PSP rise time (Figure 8B), PSP onset latency (Figure 8C), PSP decay time constant (Figure 8D), transmission failures (Figure 8E), c.v. of PSP amplitude (Figure 8F), and the inverse relationship between transmission failures and c.v. of PSP amplitude against mean PSP amplitude [Figures 8G,H, respectively; also see (Markram et al., 1997; Feldmeyer et al., 1999)].

**Figure 9:**
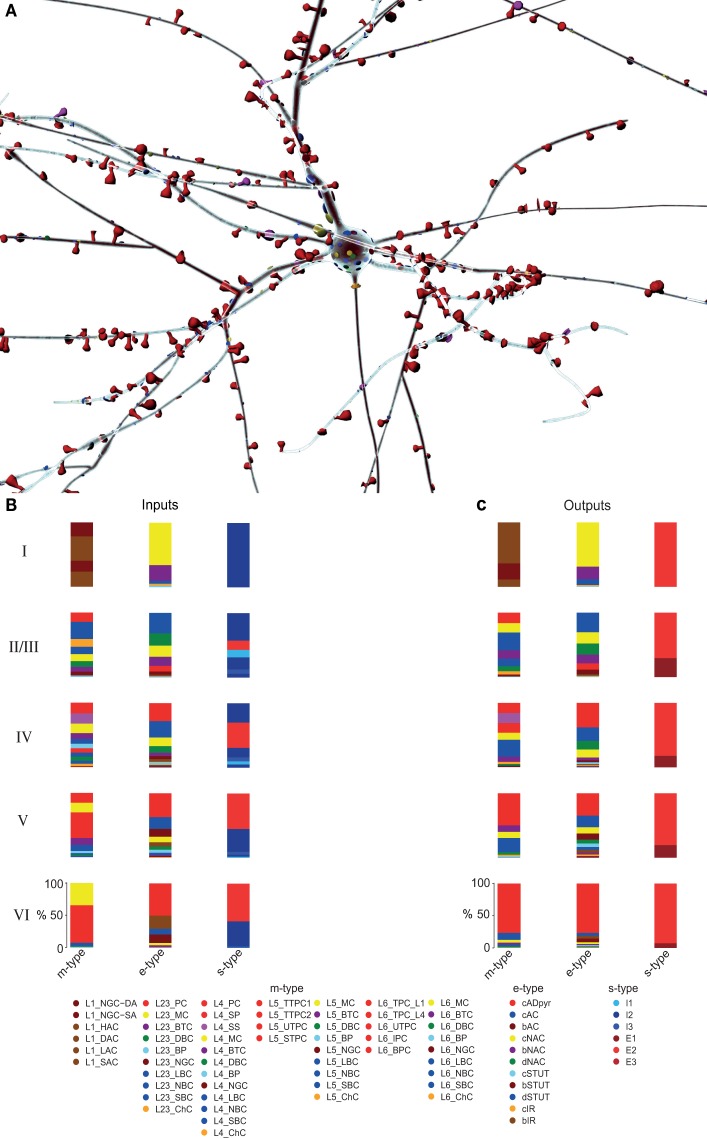
**Predicted map of afferent synaptic input to, and efferent synaptic output from an exemplar L23_PC neuron**. **(A)** Rendering of a L23_PC, predicting the map of afferent synapses color coded by m-type. **(B,C)** The intrinsic synaptic input and output, respectively from and to all m-, e-, and s-types across six layers for all L23_PCs in the reconstructed microcircuit.

The reconstruction makes it possible to predict the full complement of synaptic inputs and outputs for any given neuron. For each connection-type, a connection-type fact sheet provides predictions on the number and locations of afferent synaptic input to and efferent synaptic output from neurons belonging to specific m-types (see Figure 9A). These data categorize inputs to and outputs from all m-, e-, and s-types in the reconstructed microcircuit (see Figures 9B,C). Web links are provided to download NEURON model packages for exemplar neurons for each of the 55 m-types.

## Literature consistency

The literature consistency contains papers considered in the validation process. For each paper we identified one possible evaluation criterion. Each paper is annotated to show the evaluation criterion, and the result of the validation (consistent, possibly consistent, inconsistent). Where a paper was not actually used, it is marked as not implemented, or not applicable. Additional annotations describe the species and age of the animals used in the study, the brain region, cortical layer, and cell type concerned, the key finding, and other relevant results. The PubMed ID of the publication (PubMed is a free resource that is developed and maintained by the National Center for Biotechnology Information, at the U.S. National Library of Medicine, located at the National Institutes of Health), a web link to the publication, the name of the contact author, and the names of the persons who undertook the literature search are also provided.

The evaluation criteria are based on the morphological, molecular, electrical, synaptic, circuit anatomical, and physiological properties of the reconstructed microcircuit. For ease of presentation, papers are grouped according to the class of properties considered in their evaluation criteria.

### Morphological properties

This sub-section presents 64 papers used for the validation of experimentally reconstructed neuron morphologies. The papers provide data for Sholl statistics, dendritic and axonal lengths, dendritic and axonal volumes, soma diameters, and dendritic and axonal segment branch order and path distance statistics. Overall, the properties of the morphologies used in the reconstruction were consistent with the results reported in 17 papers, possibly consistent with 10 papers and inconsistent with nine papers. Six papers whose data were not comparable with the reconstruction (different species, different brain regions etc.) were excluded from the validation process.

### Molecular properties

During the validation process, gene expression data associated with specific neuron types was used to perform *in silico* staining. This sub-section presents six papers providing the necessary data. The papers present data on the expression of the main calcium binding proteins and neuropeptides expressed by neurons of different types.

### Electrical properties

This sub-section presents 77 papers describing the electrical properties of different cortical neuron types. Data are provided for ion channel kinetics, electrical firing patterns, and passive membrane properties, including the resting membrane potential, membrane time constant, and input resistance. Other data describe electrophysiological features including AP amplitude, AP half-width, firing frequency, inter-spike interval, and dendritic properties including bAP and EPSP/IPSP attenuation.

### Synaptic properties

This sub-section contains 81 publications used to validate the anatomical and physiological properties of synaptic connection types in the reconstruction. The data provided includes spine and bouton densities for different neuron types, as well as data for pairs of neurons belonging to specific m-types: synaptic innervation patterns, number of synaptic contacts per connection, connection probabilities, transmitter release probabilities, AMPA, NMDA, kainate, metabotropic glutamate, GABA_A_, and GABA_B_ receptor peak conductances, EPSC/IPSC properties (onset latency, amplitude, rise time, and decay time constant), EPSP/IPSP properties (onset latency, amplitude, rise time, and decay time constant), transmission failure rates, c.v. of EPSP/IPSP amplitudes, short term depression and facilitation, and tonic excitation and inhibition.

### Microcircuit anatomical properties

This sub-section presents 43 papers used to validate the anatomical properties of the reconstructed microcircuit. The properties considered include microcircuit thickness, individual layer thicknesses, layer-wise distributions and densities of neurons, total axon and dendritic lengths, total number of synapses and their densities, and layer-wise distributions of bouton densities from thalamocortical axons.

### Microcircuit physiological properties

This sub-section contains 47 papers used to assess the physiological properties of the reconstructed microcircuit. Relevant properties include the frequency of network oscillations, patterns of propagation of electrical activity across different cortical layers, the physiological properties of thalamocortical synapses, and the balance of excitatory and inhibitory synaptic conductances in different neuron types due to network activity.

## Experimental data

Over the past two decades, Markram and colleagues have experimentally characterized the cellular, and synaptic microcircuitry of developing (P13-16) rat neocortex, where the anatomical, physiological and synaptic properties are not completely mature (Markram et al., 1997; Gupta et al., 2000; Wang et al., 2002, 2004, 2006; Toledo-Rodriguez et al., 2005; Silberberg and Markram, 2007; Berger et al., 2009). The portal provides access to the data sets used in the reconstruction process, together with descriptions of the experimental protocols used to generate the data. They include:

1. Measurements of individual layer heights.
  - Data are provided in the form of microscopy images of NeuN (neuron-specific nuclear protein) stained coronal slices with annotations of individual layer extents, and spreadsheets summarizing measurements of layer heights.
2. Layer-wise distributions and densities of neurons.
  - Data are given as microscopy images of NeuN stained slices with annotations of individual layer extents, and spreadsheets summarizing measurements of neuron counts across different layers.
3. Molecular characterization of single neurons based on the expression of calcium binding proteins [parvalbumin (PV), calbindin (CB), and calretinin (CR)], and neuropeptides [somatostatin (SOM), vasoactive intestinal polypeptide (VIP), neuropeptide Y (NPY), and cholecystokinin (CCK)].
  - Data can be obtained as text files containing the expression patterns of calcium binding proteins, and neuropeptides for morphologically identified neurons.
4. More than 1000 morphological reconstructions of neurons of different m-types.
  - Data are provided as text files containing 3D representations of neuronal morphologies reconstructed using Neurolucida (neuron tracing, reconstruction, and analysis software).
5. More than 1500 electrical recordings from neurons of different e-types.
  - Data are given for current stimuli (in pA), and voltage responses (in mV) from whole-cell patch clamp recordings in single neurons acquired using IGOR Pro (scientific graphing, and data analysis software; WaveMetrics, Inc., Portland, OR, USA).
6. More than 5000 experiments from synaptically connected neurons.
  - Data are provided for post-synaptic responses obtained from whole-cell patch clamp recordings in synaptically connected pairs of neurons.

## Videos

The videos section provides movies of simulated spontaneous and evoked activity in virtual cortical slices, obtained from the reconstructed microcircuit under a variety of simulated experimental conditions. Movies of spontaneous activity show electrical activity in the reconstructed microcircuit at different levels of depolarization and extracellular Ca^2+^. Movies of evoked activity show electrical activity in response to the stimulation of thalamocortical fibers at different levels of extracellular Ca^2+^. Additional movies visualize the distribution of afferent synapses for a single exemplar neuron for each of the 55 different m-types, and predicted dendritic attenuation of EPSPs and bAPs for 5 exemplars for each of the 207 me-types.

## Tools

The tools section contains three tools used for the analysis, repair and cloning of neuronal morphologies. *NeuroM* provides a range of morphometric analyses that allow a user to quantify properties of the axonal and dendritic morphologies of neurons, and provides features that are used in the classification of different neurons into one of 55 m-types. *NeuroR* utilizes the results of *NeuroM* to identify and repair arbors that were severed during slice preparation. *NeuroC* produces clones of the neurons *repaired* by *NeuroR*, introducing statistical variations in the arbors of each cloned neuron. This procedure makes it possible to generate a limitless number of unique instances of neurons belonging to a given m-type.

## Downloads

The downloads section provides models of single neurons, synapses, and predicted maps of input-output synapses. Models are available either in the native format used in the reconstruction process, based on the NEURON simulation environment, or in the emerging NeuroML 2.0 (a model description language for computational neuroscience based on the extensible markup language–XML), and LEMS (Low Entropy Model Specification language, which aims to provide a compact, minimally redundant way of expressing models of biological systems). Models of individual neurons (in the NEURON simulation environment) can also be obtained from the me-type fact sheets [see **Morpho-Electrical Types (me-types)**]. Model packages for individual neurons contain a 3D reconstructed morphological model, models of ion channels and synapses, synaptic model parameter descriptions, and a template model of the electrical type. Additional helper scripts for the NEURON simulation environment are provided to instantiate a morphoelectrical neuron model, distribute ion channel mechanisms on axonal and dendritic arbors, and simulate electrophysiological and synaptic experiments.

## Discussion

The NMC portal was developed to serve as an online resource for experimentalists and theoreticians, allowing them to explore, analyze, challenge, and refine the reconstructed microcircuit and to derive predictions to be validated against experiments. The reconstructed microcircuit is a first draft, which will be refined in successive cycles of reconstruction-validation-experiment-reconstruction. The interactive environment provided by the NMC portal will facilitate the integration of new datasets, contributing to the refinement of the reconstruction. For example, the total length of axons in the reconstruction falls short of experimental estimates, possibly because of the difficulty of reconstructing the thin terminal axonal segments of biocytin filled neurons *in vitro*. The shortfall can be overcome by *in silico* synthesis of axons, utilizing data from *in vivo* filled neurons (see Oberlaender et al., 2011, 2012; Narayanan et al., 2015). Other examples of data that can be used to refine the reconstruction are profiles of the density of ion channels along neuronal arbors, which provide constraints for the optimization of single neuron models. Additional datasets will help to fill in biological details that are not included in the current first draft reconstruction (e.g., data on gap junctions, cholinergic modulatory mechanisms, rules for activity dependent plasticity, extrasynaptic glutamate, and GABA receptors, kinetics of metabotropic glutamate receptors, non-synaptic transmission, autaptic connections etc.). We are currently developing further data integration techniques and quality control measures, which will ensure strict compatibility of different datasets with our own experimental data in terms of species, age, cortical area and region, slice orientation and thickness, composition of extracellular and intracellular solutions, recording temperature, and technique, liquid junction potential correction etc.

The NMC Portal is part of a broad trend toward the development of large repositories of anatomical and physiological data. Other examples include NeuroMorpho, ModelDB, NeuroElectro, Open Source Brain, and the recent Allen Brain Cell Types Database. The portal contributes to this trend by integrating experimental data, models of single neurons, ion channels, synapses and detailed reconstructions of the microcircuit, and validation data in a single interactive environment. The ultimate goal is to catalyze consensus on the cellular and synaptic organization of neocortical microcircuitry (ion channel, neuron and synapse types and distributions, connectivity, etc.) and to enable community-driven refinement of the reconstruction. Newer versions of the reconstruction will be released periodically, consisting of data structures consistent with the current version. We consider that these reconstructions and the simulations they enable represent a major step in the development of *in silico* neuroscience.

### Conflict of interest statement

The authors declare that the research was conducted in the absence of any commercial or financial relationships that could be construed as a potential conflict of interest.
