# Supplementary material for: The neocortical microcircuit collaboration portal: a resource for rat somatosensory cortex
Source: Front Neural Circuits. 2015 Oct 8;9:44. doi: 10.3389/fncir.2015.00044 (PMC4597797; doi:10.3389/fncir.2015.00044)
Supplement: Supplementary file 1 [file DataSheet1.PDF]

Supplementary Material

The Neocortical Microcircuit Collaboration Portal: A Resource for Rat Somatosensory Cortex

Srikanth Ramaswamy[1,†,*], Jean-Denis Courcol[1,†], Marwan Abdellah[1], Stanislaw R. Adaszewski[1], Nicolas Antille[1], Selim Arsever[1], Guy Atenekeng[1], Ahmet Bilgili[1], Yury Brukau[1], Athanassia Chalimourda[1], Giuseppe Chindemi[1], Fabien Delalondre[1], Raphael Dumusc[1], Stefan Eilemann[1], Michael Emiel Gevaert[1], Padraig Gleeson[2], Joe W. Graham[1], Juan B. Hernando[3], Lida Kanari[1], Yury Katkov[1], Daniel Keller[1], James G. King[1], Rajnish Ranjan[1,4], Michael W. Reimann[1], Christian Rössert[1], Ying Shi[1,4], Julian C. Shillcock[1], Martin Telefont[1], Werner Van Geit[1], Jafet Villafranca Diaz[1], Richard Walker[1], Yun Wang[5,6], Stefano M. Zaninetta[1], Javier DeFelipe[7,8], Sean L. Hill[1], Jeffrey Muller[1], Idan Segev[9,10], Felix Schürmann[1] , Eilif Muller[1,*], and Henry Markram[1,4,*]

[1] Blue Brain Project, École polytechnique fédérale de Lausanne (EPFL) Biotech Campus, Geneva, Switzerland

[2] Department of Neuroscience, Physiology and Pharmacology, University College London, London, UK

[3] CeSViMa, Centro de Supercomputación y Visualización de Madrid, Universidad Politécnica de Madrid, Madrid, Spain

[4] Laboratory of Neural Microcircuitry, Brain Mind Institute, EPFL, Switzerland

[5] Key Laboratory of Visual Science and National Ministry of Health, School of Optometry and Opthalmology, Wenzhou Medical College, Wenzhou, China

[6] Caritas St. Elizabeth's Medical Center, Genesys Research Institute, Tufts University, Boston, MA, USA

[7] Laboratorio Cajal de Circuitos Corticales, Centro de Tecnología Biomédica, Universidad Politécnica de Madrid, Madrid, Spain

[8] Instituto Cajal (CSIC) and CIBERNED, Madrid, Spain

[9] Department of Neurobiology, Alexander Silberman Institute of Life Sciences, The Hebrew University of Jerusalem, Jerusalem, Israel

[10] The Edmond and Lily Safra Centre for Brain Sciences, The Hebrew University of Jerusalem, Jerusalem, Israel

[†] These authors have contributed equally to this work.

[*] **Correspondence:**

Srikanth Ramaswamy, Blue Brain Project, EPFL, Switzerland (srikanth.ramaswamy@epfl.ch)

Eilif B. Muller, Blue Brain Project, EPFL, Switzerland (eilif.mueller@epfl.ch)

Henry Markram, Blue Brain Project, EPFL, Switzerland (henry.markram@epfl.ch)

A quick guide to navigating the microcircuit

The NMC portal provides a range of navigation capabilities, described below.

Navigating the microcircuit: the user selects the microcircuit tab on the home page, where the left window represents connectivity between layers, and the thickness of the connecting ribbons represents the number of connection types between source and destination layers. The right window provides a microcircuit fact sheet showing key microcircuit properties (volume, number of neurons, number of layers etc.). A text window allows users to add annotations.

Information on a layer: the user clicks on the layer name. The portal then displays a left window showing the efferent connections of neurons in the layer, classified by m-type and a right window providing a layer fact sheet, showing the distribution of m-types, e-types and other layer properties, together with a graphical representation of the mapping between the m-types in the layer and the calcium-binding proteins and neuropeptides they commonly express. A text window allows users to annotate the sheet.

Information on an intra- or inter-laminar connection: the user clicks on the ribbon representing the connection. The portal then displays a detailed representation of the connections between neurons of different m-types in the source and destination layers-

Efferent connectivity and properties of an m-type: the user clicks on the name of the m-type. The portal displays a left window showing the connectivity, and a right window showing the morphometric properties of the m-type together with data on its constituent me-types. A *More Details* button allows users to obtain detailed statistical distributions for the morphometric properties of axons and dendrites.

Properties of an me-type. The me-type data in the m-type fact sheet contains links to separate fact sheets for each me-type. The fact sheet provides anatomical and physiological data for five exemplars of neurons belonging to the me-type, including data for the distribution of ion channels on axons, dendrites and the soma, traces of the response to step currents at 3 different intensities, an interactive 3D display of the neuron morphology, and movies showing the attenuation of post-synaptic potentials as they propagate from their dendritic location to the soma and the attenuation of action potentials as they back propagate from the soma to the dendrites. Users can download a NEURON model package for each exemplar. Again, text windows allow users to annotate the data.

Connection type data: the user clicks on the ribbon connecting source and destination m-types. The portal changes the right window to display a fact sheet for the connection-type, describing its anatomical and physiological properties. A *More Details* button allows users to obtain statistical distributions for these properties.

Supplementary Figures

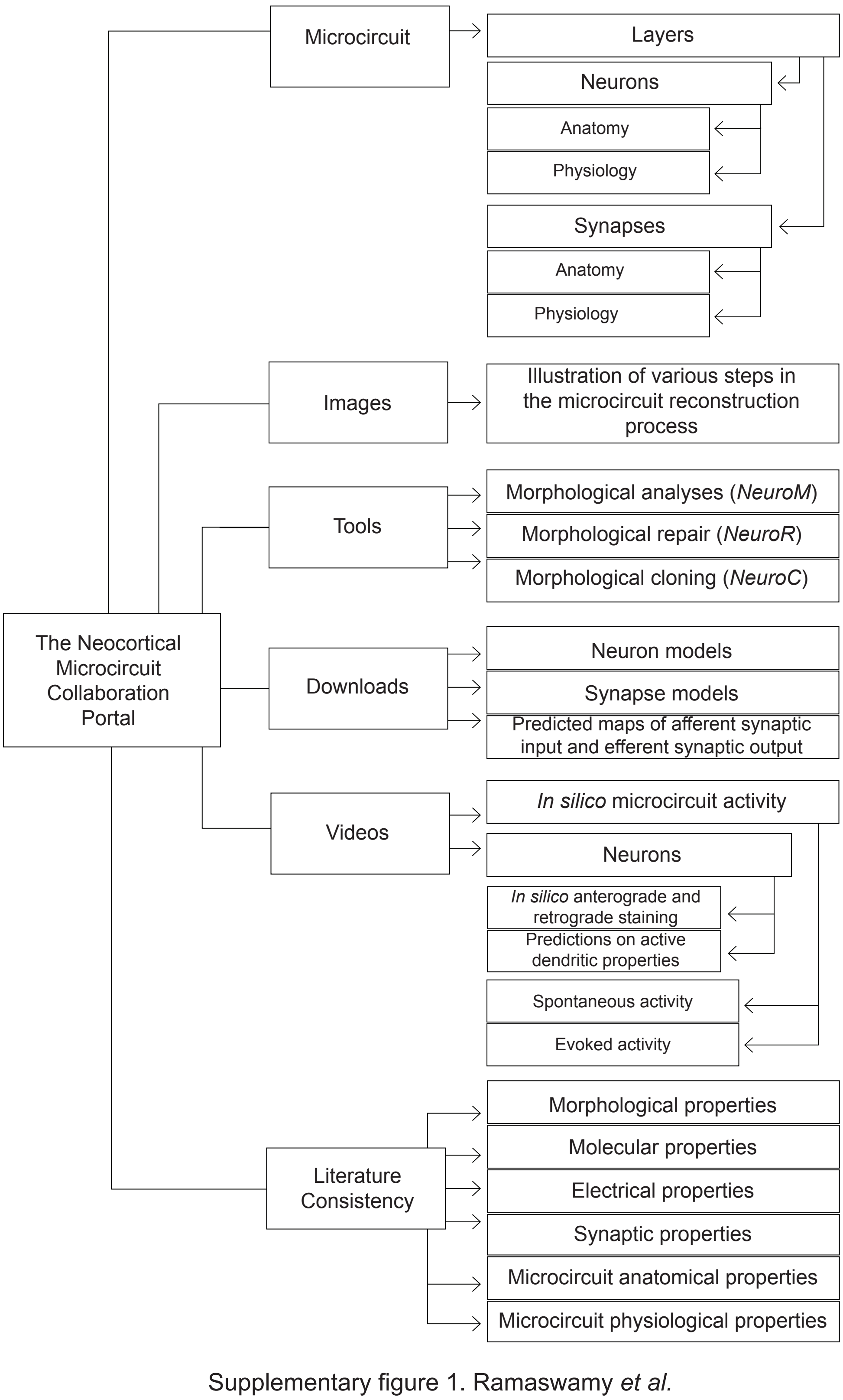

Supplementary Figure 1. The structure of the NMC portal. A tree-like overview of the NMC portal listing the different sections.

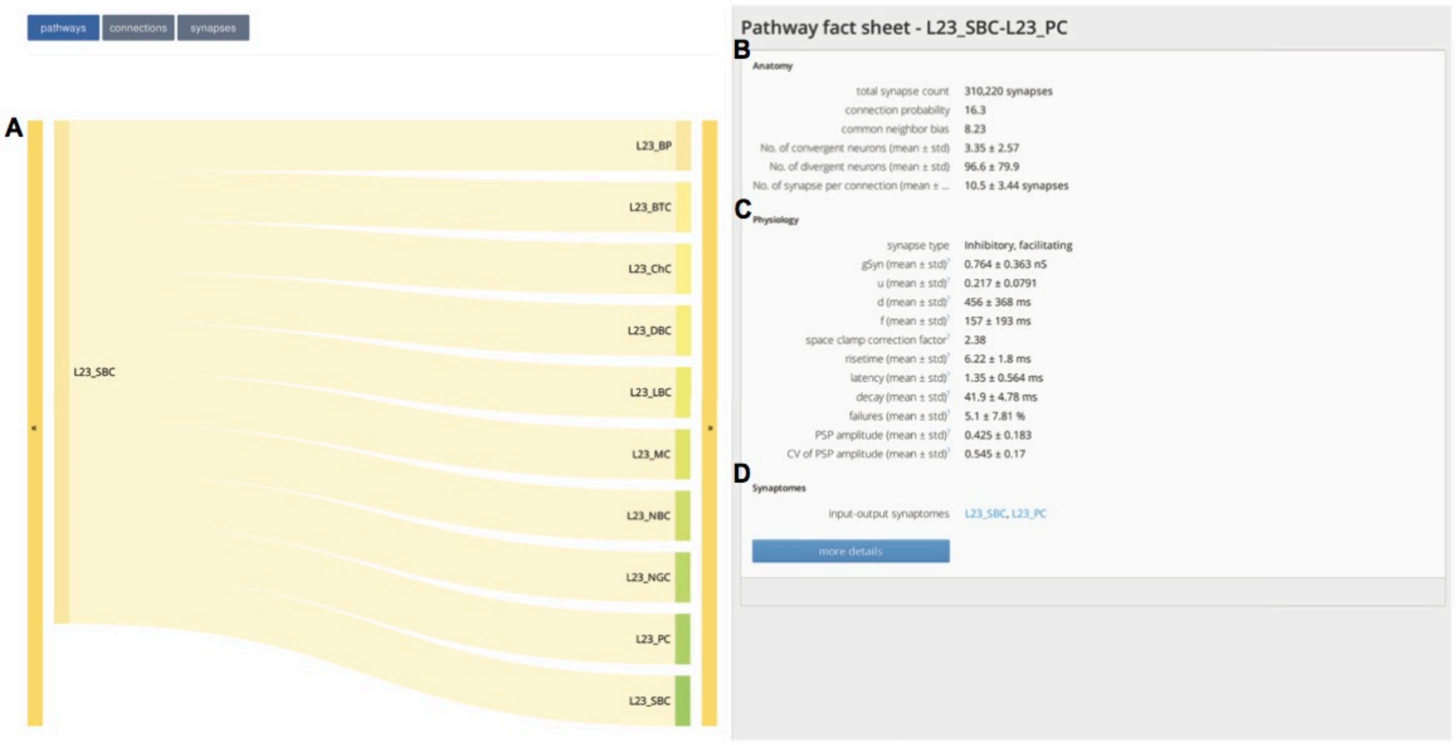

Supplementary figure 2. Ramaswamy *et al.*

Supplementary Figure 2. **Connection-type fact sheet.** (**A**) Predicted *in silico* postsynaptic targets in layer 2/3 for the L23_SBC m-type. The pathway navigator also allows direct access to relevant *in silico* fact sheets of all postsynaptic m-types. (**B**) Anatomy of all *in silico* synaptic connections from L23_SBC to L23_PC neurons. (**C**) Physiology of all *in silico* synaptic connections from L23_SBC to L23_PC neurons. (**D**) Predicted map of afferent synaptic input from and efferent synaptic output to L23_SBC, and L23_PC neurons

Supplementary Methods

The NMC portal is built on top of Liferay 6.2 Community Edition [1] bundled with Jetty 7.6 [2] and MySQL Community Server 5.1 [3]. The Liferay portal is hosted on a Red Hat Enterprise Linux 6.5 [4] virtual machine with 4 CPUs, 8 GB of memory and 100 GB of disk space. Web artifacts are built through Maven [5] and stored in a Nexus repository [6]. Deployment and server configuration are automated by Puppet [7].

The pathway navigator web component is implemented in javascript using D3.js visualization library [8] and the D3's Sankey plugin [9]. Sankey diagrams are used to visualize magnitudes of flows between nodes in the graph and serves here as a metaphor of the connectivity in the microcircuit.

The 3D morphology viewer is implemented in javascript using Three.js 3D library [10]. This view is only available to WebGL compatible web browsers. Meshes for neurites are computed in the browser based on a tree representation with segments represented as conical frustums.

The portal supports all the common evergreen browsers: Chrome, Firefox, Safari, Opera, IE10+.

EPSP and bAP attenuation movies generation process followed 3 steps:

First, design the layouts in Adobe Illustrator for various morphology types.

Second, read simulation data, target the regions of interest, render and composite in the appropriate layout.

Finally, automate and parallelize the generation of the movies. For 1000+ neurons and twice as many movies, 4 nodes – each with 12 CPUs and 3 GPUs – of a cluster were used resulting in a total render time of 54 hours.

Scientific data are integrated and synchronized into Liferay Webdav directories by Python scripts. The size of this dataset is approximately 10 GB and 42 000 files.

[1] http://www.liferay.com/

[2] http://www.eclipse.org/jetty/

[3] http://www.mysql.com/

[4] http://www.redhat.com/

[5] http://maven.apache.org/

[6] http://www.sonatype.org/nexus/

[7] http://puppetlabs.com/

[8] http://d3js.org/

[9] http://bost.ocks.org/mike/sankey/

[10] http://threejs.org/
